# Choice-architecture TB preventive therapy prescribing for HIV patients in Mozambique

**DOI:** 10.5588/pha.24.0033

**Published:** 2025-03-01

**Authors:** I. Salles, S. Munguambe, R. Chiau, E. Valverde, J.E. Golub, C.J. Hoffmann, K. Shearer

**Affiliations:** ^1^ Center for Tuberculosis Research, Johns Hopkins University, Baltimore, MD, USA;; ^2^ Eduardo Mondlane University, Maputo, Mozambique;; ^3^ Fundação Aurum, Maputo, Mozambique.

**Keywords:** acceptability, healthcare workers, qualitative research, default prescribing, behavioral economics, TPT, CAT

## Abstract

**INTRODUCTION:**

Despite the effectiveness of TB preventive treatment (TPT) in reducing TB incidence and mortality among people living with HIV (PLHIV), uptake has been low. We conducted a cluster randomised trial to evaluate a choice architecture-based intervention for prescribing TPT (the ‘CAT’ study) to PLHIV in Mozambique, nested within the short-course 3HP regimen roll-out, and qualitatively assessed intervention acceptability and feasibility with healthcare workers (HCWs).

**METHODS:**

The CAT intervention comprised training on default TPT prescribing and prescribing stickers integrated into antiretroviral therapy (ART) stationery. We assessed intervention acceptability and feasibility to increase TPT prescribing through 25 in-depth interviews (IDIs) with HCWs from participating clinics between August and September 2022. Thematic analysis of the IDIs identified key themes.

**RESULTS:**

Participants reported a positive impact of the intervention on patient care, though workload opinions varied. Participants reported that CAT did not significantly alter routine TPT prescribing processes but highlighted the need for reminders and decision-support tools. CAT was viewed to streamline patient management, particularly identifying eligible TPT patients and simplifying documentation.

**CONCLUSION:**

The CAT strategy could enhance TPT delivery to PLHIV and integrate it into preventive care for other diseases.

TB is a leading cause of death for people living with HIV (PLHIV).^1^ TB preventive treatment (TPT) reduces the risk of TB by over 30% among PLHIV;^2–4^ yet, delivery of TPT has historically been low. This ‘science to service gap^’^ – the difference between knowledge generated from research and clinic-level service delivery – is reflected in TPT delivery to 46% of the estimated 39 million PLHIV globally.^1,5,6^ Success or failure of service delivery can occur for multiple reasons and at multiple levels, including the level of buy-in from key stakeholders, reflected by national policy and funding support, and from clinicians and other clinical staff.^6^

Increased TPT utilisation in Mozambique is a priority of the Ministry of Health.^1^ TB incidence in Mozambique was 361/100,000 in 2022, with 25% of TB cases occurring in PLHIV.^7,8^ Under-delivery of TPT is often a result of multiple factors, including clinician hesitancy to prescribe due to perceptions of risk associated with TPT, lack of confidence in the appropriate prescribing process, and remembering to prescribe at the appropriate time.^9–13^ Due to under-delivery, missed opportunities for TPT initiation and continuation compromise TB prevention efforts.^14^

The WHO recommends TPT for all PLHIV. Asymptomatic PLHIV should receive TPT, while symptomatic individuals should initiate treatment after excluding TB disease.^15^ In Southern African clinics, 5–10% of PLHIV initiating antiretroviral therapy (ART) have TB disease,^16,17^ with some guidelines also excluding pregnant individuals. Thus, 80–90% of PLHIV are expected to qualify for TPT, allowing providers to focus decision-making on the minority of cases with contraindications.

To address the gap in TPT prescribing to PLHIV, we designed a choice architecture-based intervention targeted at healthcare workers (HCWs) to make TPT prescribing the default option. Choice architecture is a behavioral economics theory that posits how choices are presented and how these influence the individual’s decision-making process.^18,19^ Choices requiring active decision-making take more cognitive effort and are less likely to be selected. Conversely, choices that require less decision-making – such as the default if no action is taken – are more likely to be accepted.^20,21^ We used choice architecture principles to develop an intervention to shift provider decision-making from deciding who is eligible for TPT to assuming all PLHIV are eligible, redirecting cognitive effort and decision-making to identifying the smaller subset with a specific contraindication (e.g., symptoms necessitating TB investigation),^22,23^ shifting the burden to justifying non-prescription of TPT.

We tested the intervention in a cluster-randomised trial (the ‘CAT’ study) conducted in Mozambique, Malawi, and Zimbabwe, nested within the IMPAACT4TB project.^24^ IMPAACT4TB was a multi-component project that supported the roll-out of a 3-month weekly isoniazid-rifapentine (3HP) as an alternative to the standard 6-month daily isoniazid (6H) regimen in 12 high TB burden countries.^25^ Here, we used qualitative methods to evaluate the acceptability and feasibility of the CAT intervention at the clinic level in Mozambique.

## METHODS

The choice architecture intervention comprised clinician training on the default prescribing approach and was supported by a TPT prescribing sticker to consider TPT part of every ART prescribing or re-prescribing visit. Using the stickers, HCWs could indicate the TPT regimen (6H or 3HP), the prescription date, and the number of months dispensed (Figure). Stickers were placed on patient cards, part of routine care, to document all prophylaxis and clinic visits, remind HCWs to prescribe TPT and facilitate its integration into daily clinical practice.

**FIGURE.**
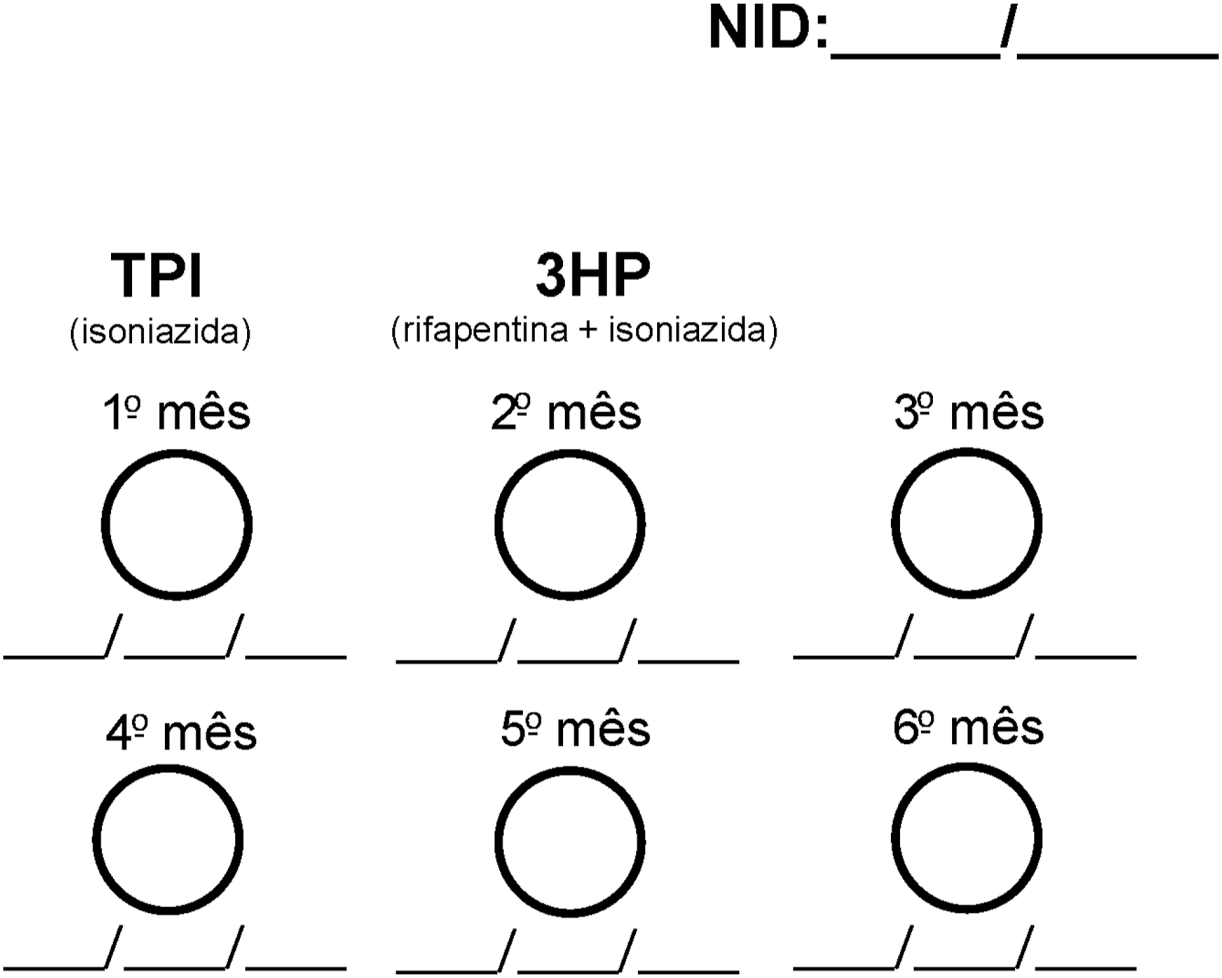
TPT prescribing sticker. TPI = *terapia preventiva com isoniazida* (isoniazid preventive therapy); 3HP = 3-month weekly isoniazid-rifapentine; TPT = TB preventive therapy.

Between August and September 2022, in-depth interviews (IDIs) were conducted with a total of 25 HCWs providing HIV-related care at study clinics for at least three months. HCWs were purposively selected and interviewed at all 10 intervention and 10 control clinics across three districts of Mozambique: Mandlakazi, Chokwe, and KaMavota. IDIs were conducted using a semi-structured interview guide composed of open-ended questions. The interviews were conducted in Portuguese, audio-recorded and transcribed verbatim for in-depth analysis using NVivo 14.0 (Lumivero, Denver, CO, USA).

Coding categories were evaluated and transformed into potential themes based on the recurring patterns in responses. Two study team members independently coded the transcripts and met regularly to compare emergent themes. These themes were compared across groups and regional contexts and cross-verified with study investigators with first-hand knowledge of the field dynamics. Salient themes were extracted, and quotes were selected to illustrate key findings.

All study participants provided written informed consent. Interviews were conducted in a private setting at their respective clinics. The study protocol was approved by the Institutional Review Board (IRB) of the Johns Hopkins University School of Medicine, Baltimore, MD, USA (IRB00227388); and the Mozambique Comité Nacional de Bioética em Saúde, Maputo, Mozambique (CNBS, 359/CNBS/2022).

## RESULTS

### Sample characteristics

Twenty-five interviews were conducted with HCWs, with a balanced distribution between intervention (*n* = 13) and control (*n* = 12) arms. Most participants were female (64%), the mean age was 32.9 years (range 24–63), and the majority (60%) were General Medicine Technicians (mid-level providers, equivalent to Physician Assistants). Eight (32%) participants were Maternal and Child Health Care (MCH) Nurses, one was a Medical Agent (basic-level provider, equivalent to Medical Assistant), and one was a Medical Doctor. Nearly half (48%) of the participants had one to four years of experience in the HIV-TB sector, while approximately one-third (36%) had over 4 years of experience (Table).

**TABLE.** Sociodemographic characteristics of the sample.

Characteristic	Total (*n* = 25) *n* (%)	Intervention Arm(*n* = 13) *n* (%)	Control Arm(*n* = 12) *n* (%)
Sex			
Female	16 (64)	8 (62)	8 (67)
Male	9 (36)	5 (38)	4 (33)
Age, years, mean (range)	32.9 (24–63)	32.7 (24–40)	33.2 (25–63)
Job category			
General Medicine Technician	15 (60)	9 (69)	6 (50)
Maternal and Child Healthcare Nurse	8 (32)	3 (23)	5 (42)
Medical Agent	1 (4)	1 (8)	0
Medical Doctor	1 (4)	0	1 (8)
Time of experience in HIV-TB sector, years			
<1	4 (16)	3 (23)	1 (8)
1–4	12 (48)	6 (46)	6 (50)
>4	9 (36)	4 (31)	5 (42)

### TPT prescribing part of routine HIV treatment initiation package

HCWs viewed TPT prescribing for PLHIV as part of their duties and considered it routine before the introduction of the CAT intervention. Providers were confident in TPT prescribing for PLHIV and using the CAT intervention to facilitate the process. However, TPT prescribing using CAT did not significantly deviate from existing TPT prescribing practices (standard of care).

I feel confident…it (TPT prescribing) became our daily bread. So, this is already inside us, we sleep with it, we wake up with it. It’s already in our minds. I feel good about it. I feel confident. (General Medicine Technician, 27 years, male, Intervention Arm)

The prevailing mindset that clinical evaluation must precede TPT provision remained when HCWs described their prescribing approach with CAT, indicating a challenge in embracing the default prescribing model.

The patient doesn’t just arrive at the clinic to receive medication (TPT) before evaluation, no, the evaluation is first, then the clinician decides whether they meet criteria or not. If they do, let’s give it, if not then there is no need. (General Medicine Technician, 29 years, male, Intervention Arm)

CAT was seen as an enhancement to existing practices rather than altering practices. However, eliciting clear descriptions from participants about how CAT enhanced and did not change practices was challenging. The following quote suggests that CAT did not introduce significant changes to daily routine; however, participants expressed that CAT improved care by increasing TPT prescription rates.

CAT didn’t change our routine, it actually improved since we also increased the prescription rates, for 3HP and 6H (General Medicine Technician, 40 years, male, Intervention Arm)

### Reliance on eligibility criteria and the role of effective reminder systems

Although TPT prescription was considered part of the routine care package for PLHIV, not all HCWs readily recalled the criteria for its administration, such as contra-indications. Newer HCWs relied heavily on reminders or reference material to ascertain TPT eligibility. A medical doctor from the control arm highlighted HCWs’ difficulty remembering TPT prescribing criteria, emphasising the inherent complexities of TPT prescribing and the value of effective reminder systems to support HCWs when making accurate eligibility determinations.

Many times, we don’t have that (TPT criteria) in mind, but since it (TPT prescribing algorithm) is here (consultation room), we consult the reminder. (Medical Doctor, 63 years, male, Control Arm)

An HCW noted that a lack of knowledge about the criteria could lead to missed opportunities to offer TPT to eligible patients. This also may have reflected the lack of internalisation of the principles of default TPT prescribing and withholding TPT unless the patient met the eligibility criteria.

I think to increase prescriptions (TPT), we should know more about the criteria, because if we don’t know the criteria, we won’t offer them to our patients. (General Medicine Technician, 28 years, female, Intervention Arm)

The CAT sticker was a practical tool for HCWs to guide prescribing TPT at patient visits. HCWs emphasised the value of this simple reminder.

(CAT) made it easier... there was a time when we would miss patients who met the criteria (to receive TPT) because of the lack of reminders. With the entry of these reminders, thank God. (General Medicine Technician, 27 years, male, Intervention Arm)

Heavy patient caseloads were seen as challenging in assessing patients for TB and TPT eligibility. HCWs expressed that more patients were evaluated for TB and TPT eligibility using CAT. Since TPT prescriptions were documented on the stickers, HCWs had this information readily visible to prompt them to consider TB and TPT at every visit.

Basically, it’s like this, people don’t go unnoticed regarding tuberculosis. Because not always, not every clinician has the patience, because one clinician is responsible for 50 patients. It’s not easy, and every patient who comes to the clinic is evaluated for tuberculosis, so it’s to be commended. (MCH Nurse, 35 years, female, Intervention Arm)

Referring to the card and marking the TPT prescription on the sticker during each month of treatment aided in tracking and scheduling TPT administration, allowing HCWs to quickly identify when treatment would be completed, streamlining the overall management of TPT for patients.

It always reminds us…. It (the sticker) serves as a reminder for us when the patient comes in, so we receive the card and then we remember that ahh this patient also must do it (TPT) so we cross it off there and we can identify the end of the month when the prophylaxis will end too. It’s important because it serves as a reminder for us. (MCH Nurse, 29 years, female, Intervention Arm)

CAT was seen as effectively minimising the risk of overlooking eligible patients, improving the prescribing process. The tool was a reliable reminder, ensuring appropriate care and effective tracking of TPT prescribing and completion. This reaffirmed the importance of reminder systems in supporting HCWs in providing optimal care to patients needing TPT.

### Benefits to patient management

HCWs noted that patient records and forms used to assess for TPT eligibility and monitor treatment progress may have missing information, and key information can be challenging to locate.

What must happen is more and more improvement, improvements to filling out the records, reporting all the data, for all the required information to be clearer in the record itself. Because at some point you can pick up a record and discover it has information that was not filled in, but when you pick up the patient’s follow-up form, you find the information that was not here (in the record) but it exists there (in the follow-up form), so everything must be coordinated. (General Medicine Technician, 32 years, male, Control Arm)

Respondents underscored that documentation using CAT was comprehensive, as it supported initial eligibility assessment, monitoring medication re-prescriptions during the treatment course, and determining TPT completion.

Thank God we now have something so important, the stickers. At least they (the cards) have the stickers, making it easier. All the patients we evaluate and put on (TPT), we paint a circle (on the sticker). This makes it easier for us to follow (the patient’s treatment) or decide on TPT initiation. Because there are patients that at the beginning (ART initiation) didn’t initiate TPT. (General Medicine Technician, 27 years, male, Intervention Arm)

Respondents noted that CAT was comprehensive because it provided a reminder to assess patient eligibility for TPT at ART initiation and follow-up visits for those already on ART who had never completed TPT.

It (sticker) facilitated, so that we were aware if the patient had or had not done 3HP or 6H. We won’t initiate a process the patient previously finished. (General Medicine Technician, 40 years, male, Intervention Arm)

Opinions regarding CAT’s impact on workload were mixed. Given the similarity in CAT prescribing procedures to the standard of care, some HCWs perceived CAT as an unnecessary addition to their workload. An MCH Nurse suggested that CAT was redundant because patient records already tracked the same information as the CAT stickers.

The patient record already shows…if the patient has finished or has just started or is continuing with prophylaxis. (MCH Nurse, 31 years, female, Intervention Arm)

However, most HCWs indicated that the CAT approach facilitated TPT documentation and simplified identifying patients who had already been prescribed TPT using CAT.

The stickers make it easier to understand, they facilitate determining if the patient has already done (TPT)...the stickers and cards make it easier. (General Medicine Technician, 37 years, female, Intervention Arm)

Respondents widely agreed that the CAT approach allowed them to provide superior patient care for TB prevention and general health needs. The following quote highlights CAT’s benefit to patient management and follow-up related to broader healthcare needs.

It will help us to better control our patients, so we can offer better follow-up. (General Medicine Technician, 40 years, male, Intervention Arm)

With the patient’s TPT history readily accessible through the CAT sticker, HCWs remembered to continue to prescribe TPT at follow-up visits, fostering better continuity of care.

I feel good, because we have a way of always remembering it (TPT). I always remember it through the information recorded in these documents (the card and sticker). This takes away the possibility of a patient escaping. For example, they started last month (TPT) and they can’t take it now because maybe I got confused and forgot, but this document makes it easier because they come for the second-month appointment and must leave with the medicine. (General Medicine Technician, 32 years, male, Intervention Arm)

HCWs recognised CAT’s role in streamlining patient management from TPT initiation through follow-up, aiding in consistent prescribing throughout the treatment course.

## DISCUSSION

There was high acceptance and support for the CAT intervention; however, it appeared to be perceived as enhancing workflow rather than shifting to true opt-out TPT prescribing. HCWs acknowledged the efficiencies introduced by CAT, yet they remained anchored to established practices prioritising eligibility assessments over default prescribing, potentially reflecting an ingrained clinical framework that emphasises risks rather than benefits of TPT.

The CAT approach – the supporting sticker and training – heightened awareness of TPT’s value and facilitated access to patient-level information. Centralised TPT history documentation on the CAT stickers streamlined eligibility assessments and supported continuity in TPT prescribing at follow-up visits. Despite CAT not altering the prescribing approach, its utility as a reminder and documentation tool aligns with reducing cognitive load and reinforces findings from other choice architecture intervention studies in TPT prescribing.^22,23^ This effect may promote consistent TPT application by streamlining documentation requirements, addressing reported barriers to TPT and implementing choice architecture.^26,27^ This underscores the value of easily accessible and complete records for supporting TPT prescribing and treatment monitoring.

The goal was to routinise prescribing for most PLHIV, changing the focus from identifying eligible individuals to assuming eligibility for all PLHIV unless clear contraindications existed, focusing cognitive effort on the minority with contraindications. While the introduction of 3HP alongside CAT was favorably received, signaling a positive transition to short-course TPT regimens, HCWs did not fully comprehend the introduction of 3HP and CAT as separate initiatives. Consequently, CAT was primarily viewed as a documentation tool rather than a re-framed approach to TPT prescribing. This misunderstanding likely contributed to perceptions of CAT as resembling standard care without notably reducing workload, perhaps stemming from the nature of training and clinical mentoring, which may not have sufficiently achieved acceptance of the default approach.

Research indicates that user-perceived effectiveness of a default choice increases acceptance.^28,29^ Improved training that communicates the benefits of default TPT prescribing over standard care and emphasises this conceptual shift could increase acceptance of CAT as the preferred strategy for TPT delivery, ultimately increasing TPT prescribing for PLHIV.

Behavioral economics suggests cognitive biases cause individuals to prefer default options that require less cognitive effort, particularly in high-cognitive load environments like healthcare, where frequent decision-making can cause fatigue.^20^ Choice architecture interventions have shown success across healthcare^28^ – such as opt-out HIV testing,^30,31^ colorectal cancer screening,^32^ and vaccination standing orders^33^ – demonstrating how default options can streamline decision-making and enhance preventive care delivery. These interventions, like the CAT sticker, can simplify complex workflows, reducing the cognitive burden on providers in high-demand settings.

The overburdened and under-staffed health system in Mozambique mirrors those of many high TB burden settings. The findings demonstrate the application of CAT in routine clinical practice, including the challenges associated with introducing a novel tool into established healthcare routines in resource-limited settings. This realistic setting underscores the robustness of the findings, as the intervention demonstrated high acceptability among HCWs and appeared to facilitate care in contrast to many attempts at practice innovation that may add to workload and distract from other clinician responsibilities.

Results from the CAT cluster randomised trial indicated a ‘signal’ for increased TPT prescribing to new ART clients in Mozambique. Although the trial was underpowered to detect a statistically significant difference between intervention and control facilities at the country level, these findings suggest potential benefits of the CAT intervention in enhancing TPT uptake.^34^

While the positive reception of CAT in Mozambique provides insights into the potential benefits of its integration in similar settings, local healthcare capacities and dynamics must be considered to ensure successful implementation. The qualitative approach uncovered contextual and behavioral factors affecting TPT uptake, illustrating how environmental and cognitive elements intersect.

Functioning as a robust reminder and simplifying administrative tasks, the CAT approach enhances TPT administration and monitoring for PLHIV, supporting a comprehensive healthcare approach and improving the quality of life for PLHIV.

Integrating routine practices with innovative tools may aid in increasing TPT prescribing. A default prescribing strategy could be a valuable tool in the ongoing efforts to improve TPT prescribing within this high-risk group and has the potential for integration into other prophylactic medications.
